# Effect of agro-ecological zone, season of birth and sex on pre-weaning performance of Nguni calves in Limpopo Province, South Africa

**DOI:** 10.1007/s11250-016-1179-2

**Published:** 2016-11-04

**Authors:** T. J. Mpofu, M. M Ginindza, N. A. Siwendu, K. A. Nephawe, B. J. Mtileni

**Affiliations:** 1 Department of Agricultural Economics and Animal Production, University of Limpopo, Private Bag X1106, Sovenga, 0727 South Africa; 2 Department of Animal Science, Tshwane University of Technology, Private Bag X680, Pretoria, 0001 South Africa

**Keywords:** Weaning weight, Pre-weaning average daily gain, Pre-weaning gain

## Abstract

The study was conducted to determine the effect of agro-ecological zone, season of birth and sex on Nguni calves’ pre-weaning performance. Production indices such as birth weight (BW), weaning weight (WW), pre-weaning average daily gain (P-ADG) and pre-weaning gain (P-WG) were assessed in the different agro-ecological zones. Herd records on performance of 826 Nguni calves’ from nine Nguni herds representing different agro-ecological zones: arid zone (*n* = 217); semi-arid zone (*n* = 296); dry sub-humid zone (*n* = 118) and humid zone (*n* = 195) were used for the analysis of pre-weaning calf performance. General linear model (GLM) procedure of SAS (2013) was used to analyse data, whereas mean separation was conducted using Tukey’s HSD test. Agro-ecological zone had a great influence (*P* < 0.01) on performance levels arising from pasture conditions which were dependent on rain, temperature, topography and soil type. Fluctuations in WW, P-ADG and P-WG performance across agro-ecological zones depicted the sensitivity of Nguni calves’ to postnatal stress. Calves’ in humid zone had higher performance with 121.21 kg for WW, 96.83 kg for P-WG and 0.477 kg/day for P-ADG. The lowest WW (114.51 kg), P-WG (89.98 kg) and P-ADG (0.438 kg/day) were observed in arid zone. Male calves were heavier at weaning (128.18 kg), P-ADG (0.503 kg/day) and total gain (103.03 kg); however, similar BW of 25 kg was observed for both male and female calves. Season had a significant (*P* < 0.05) effect on BW, P-ADG and P-WG. The P-ADG was 0.461 kg/day for calves born in summer and 0.449 kg/day for calves born in winter season. Calves born in summer gained 94.69 kg and calves born in winter gained 92.10 kg. Summer calves gained 2.59 kg more than winter calves. Summer heifer calves performed poorly whilst summer male calves outperformed heifer calves in terms of WW, P-WG and P-ADG. Pre-weaned calves in humid zone outperformed all calves in other agro-ecological zones. It was concluded that acceptable levels of growth are achievable from Nguni cattle under the different agro-ecological zones of Limpopo province, South Africa.

## Introduction

Growth of an animal is influenced by genetic and non-genetic factors (Bourdon 2000). The importance of growth rate in beef cattle production has received ample attention from various researchers (Arthur et al. 2001; Lawrence et al. 2002). The pre-weaning growth performance of a calf is one of the most important factors for beef productivity (Correa et al. 2006). Reduced growth performance is the major limiting factor amongst other factors of cattle production in the tropics (Jones and Hennessy 2000); however, challenges placed on production traits are mainly environmentally related (Duarte-Ortuno et al. 1988; Howden et al. 1999). Mekonnen and Goshu (1996) reported that traits such as birth and weaning weight as well as growth and survival to weaning have important implications on herd productivity.

Growth rate of livestock is influenced by several factors; these include production systems, breed, age, sex, nutritional level, hormonal status and environment (Owens et al. 1993; Moyo 1996). Production environments vary as a result of different management practices and constantly changing climatic conditions leading to variability in animal performance. An animal of good genetic value may perform poorly when the production environment is not favourable due to the negative interaction between the animal’s genes and its environment (Botsime 2006).

Agro-ecological zone as described by rainfall, temperature, vegetation type, soil type and topography is a significant source of variation in pre-weaning production performance (Bufenning et al. 1982; Dooley 1982; Ronchiotto 1993). Season of birth, the interaction between sex and season of birth and the interaction between agro-ecological region and season of birth are important sources of variation in growth of beef cattle (Botsime 2006). Keeping animals that are in harmony with the environment in which they are maintained result in the maximum utilisation of natural resources. Birth weight (BW), weaning weight (WW), pre-weaning average daily gain (P-ADG) and pre-weaning gain (P-WG) are several measures of calves’ pre-weaning production performance. There is currently a paucity of information on the Nguni calves pre-weaning production performance in different agro-ecological zones. This research was therefore conducted to determine the effect of agro-ecological zone, season of birth and sex on Nguni calves pre-weaning performance in Limpopo province.

## Materials and methods

**Fig. 1:**
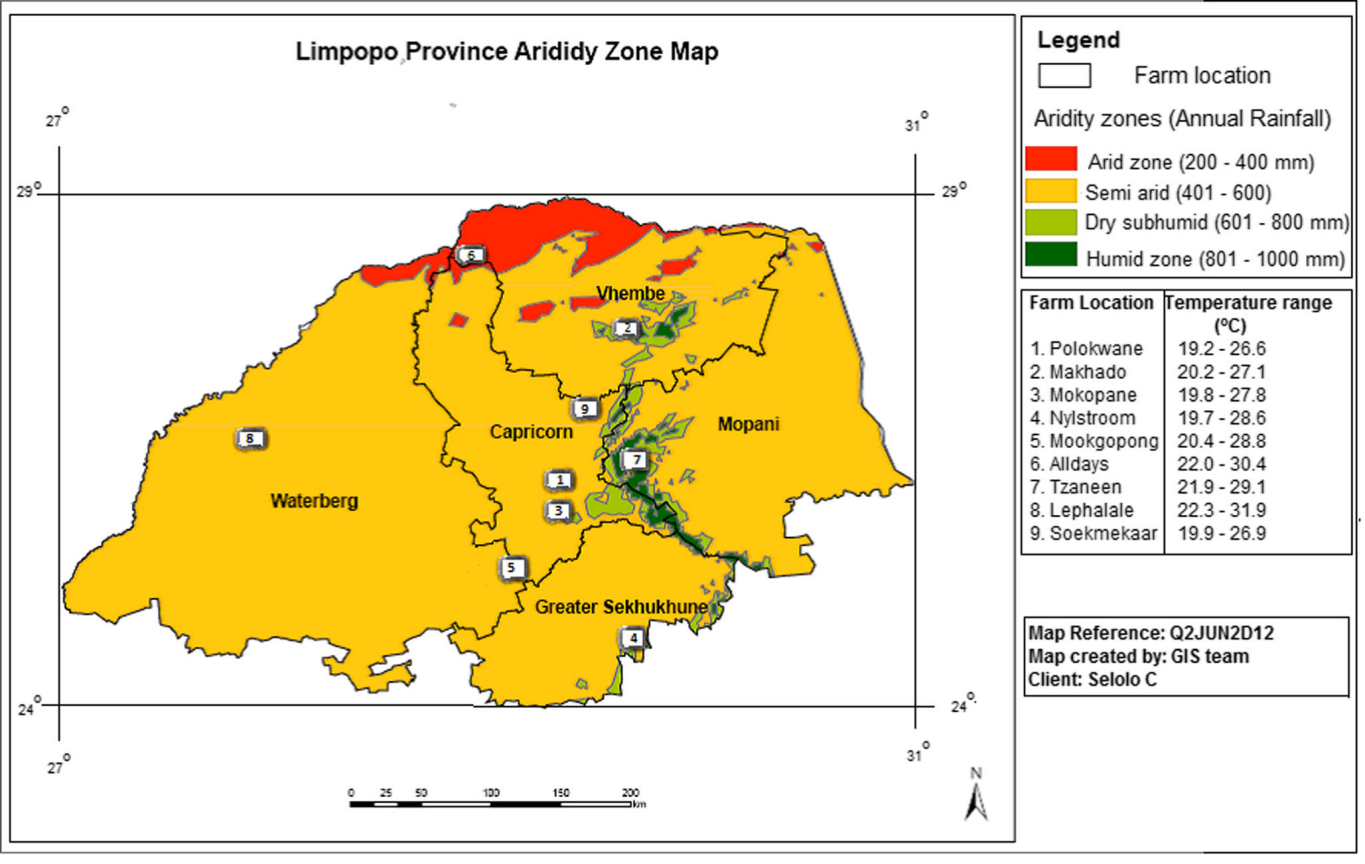
Map representing the selected nine Nguni herds representing four agro-ecological zones of Limpopo Province, South Africa

Secondary herd records on 826 Nguni calves from 2008 to 2013 obtained from nine Nguni herds representing four different agro-ecological zones were used in this study (Fig. 1). The agro-ecological zones were as follows: arid zone (*n* = 217); semi-arid zone (*n* = 296); dry sub-humid zone (*n* = 118) and humid zone (*n* = 195). The selected agro-ecological zones vary in rainfall distribution, temperature, major topographic features, vegetation type and farming systems (Table 1).

**Table 1:** Agro-ecological zones and their features in Limpopo Province, South Africa

Eco-zone	Location	Veld type	Prevailing grass species
Arid	Alldays	Sweet	*Cenchrus ciliaris*, *Panicum maximum*
	Lephalale	Mixed	*Aristida tranvaalenesis*, *Panicum maximum*
	Polokwaane	Mixed	*Eragristis curvula*, *E. capensis*
Semi-arid	Mokopane	Sourveld	*Eragristis curvula*, *E. capensis*
	Nylstroom	Sourveld	*Panicum maximum*, *Themeda triandra*
	Mookgopong	Sourveld	*Panicum maximum*, *Themeda triandra*
	Soekmekaar	Mixed	*Eragristis curvula*, *Themeda trianda*
Dry sub-humid	Makhado	Sweet	*Panicum maximum*, *Eragrostis Frichophora*
Humid	Tzaneen	Sourveld	Cymbopogon caesius, Themeda trianda

All selected herds were kept under extensive grazing system. Herds comprise of one bull and thirty (30) females were made for breeding purposes. Cows were exposed to bulls for limited breeding period of 90 days in two breeding seasons (Table 2). All calves were weaned simultaneously when the last calf reached 205 days of age. No supplementary feeding or licks was supplied.

**Table 2:** Classification of seasons according to months of birth

Season	Sate of vegetation	Month of birth
Summer	Green pastures	November
		December
		January
Winter	Dry pastures	May
		June
		July

P-ADG, P-WG and corrected weaning weight were determined using the following equations:

$$ \mathrm{P}-\mathrm{A}\mathrm{D}\mathrm{G}=\frac{\mathrm{Weight}\ \mathrm{gain}\ \left(\mathrm{kg}\right)}{\mathrm{Days}\ \mathrm{in}\ \mathrm{trial}\ \left(\mathrm{days}\right)} $$

$\mathrm{Corrected}\;\mathrm{W}\mathrm{eaning}\;\mathrm{weight}=\mathrm{B}\mathrm{W}+a\;\left(\frac{\mathrm{Actual}\;\mathrm{W}\mathrm{W}-\mathrm{B}\mathrm{W}}{\mathrm{Age}\;\mathrm{in}\;\mathrm{days}\;\mathrm{at}\;\mathrm{weaning}}\right)\times 100$ (Szabo et al. 2012)

where *a* = constant (205 days)

BW: birth weight
WW: weaning weight

$$ \mathrm{P}-\mathrm{W}\mathrm{G}=\mathrm{Corrected}\ \mathrm{weaning}\ \mathrm{weight}\ \left(\mathrm{kg}\right) - \mathrm{B}\mathrm{W}\ \left(\mathrm{kg}\right) $$

Performance data was analysed using the PROC GLM procedure of SAS (2013). Factors fitted in the model included agro-ecological zone, calving season, sex of calf, agro-ecological zone × season interactions and agro-ecological zone × sex of calf interactions. Tukey’s honestly significant difference (HSD) test was used to test for significant differences between treatment means (*P* < 0.05). The following model was used:

$$ {\mathrm{Y}}_{\mathrm{ikl}} = \mu + {\mathrm{H}}_{\mathrm{j}} + {\mathrm{S}}_{\mathrm{k}} + {\mathrm{T}}_{\mathrm{l}} + {\mathrm{H}\mathrm{S}}_{\mathrm{j}\mathrm{k}} + {\mathrm{H}\mathrm{T}}_{\mathrm{j}\mathrm{l}} + {\mathrm{H}\mathrm{S}\mathrm{T}}_{\mathrm{ikl}} + {\mathrm{e}}_{\mathrm{ijk}} $$

where

Y_ikl_: Observations on BW, WW, Pre-weaning ADG and P-WG
*μ*: Underlying constant common to all observations
H_j_: Fixed effect of agro-ecological zone
S_k_: Fixed effect of season of birth
T_l_: Fixed effect of sex of calves
HS_jk_: Agro-ecological zone × season interactions
HT_jl_: Agro-ecological zone × sex of calf interactions
HST_ikl_: Agro-ecological zone × season × sex interactions
e_jkl_: Random residual/error

## Results

Sex of calves was a significant (*P* < 0.05) source of variation for WW and P-WG; however, BW and P-ADG were not affected (*P* > 0.05) by sex of calves (Table 3). Male calves recorded higher WW (128.18 kg), P-ADG (0.503 kg/day) and P-WG (103.03 kg) than heifer calves.

**Table 3:** Effect of sex of calves on pre-weaning calf performance (kg) of Nguni cattle in Limpopo province, South Africa

Source	Birth weight (kg)	Weaning weight (kg)	P-ADG (kg/day)	P-WG (kg)
Female	24.89^a^ ± 0.2025	108.62^b^ ± 0.8994	0.413^b^ ± 0.1283	83.73^b^ ± 0.9207
Male	25.15^a^ ± 0.2042	128.18^a^ ± 0.9069	0.503^a^ ± 0.1294	103.03^a^ ± 0.9283

^a, b^Column means with different superscripts differ significantly (*P* < 0.05)

The interaction between agro-ecological zone and sex of calves is presented in Table 4. Male calves in arid zone were lighter (123.42 kg) at weaning whilst male calves in semi-arid were heavier (128.78 kg) than all male calves throughout agro-ecological zones. Female calves in humid zone were lighter at birth (23.42 kg) whilst female calves from dry sub-humid zone were heavier (24.58 kg) than all female calves throughout agro-ecological zones. However, female calves in arid zone performed poorly (WW = 105.60 kg, P-ADG = 0.394 kg/day, P-WG = 80.69 kg) whilst male calves in humid zone performed better (WW = 131.66 kg, P-ADG = 0.514 kg/day, P-WG = 106.34 kg) than all calves throughout the agro-ecological zones. This also shows that whatever the agro-ecological zone, male calves yielded better performance than their female contemporaries on the variables studied.

**Table 4:** Agro-ecological zone and sex of calves’ interaction effects on pre-weaning calf performance (kg) of Nguni cattle in Limpopo province, South Africa

Source	*N*	BW	WW	P-ADG	P-WG
Arid × female	114	24.91^bcd^ ± 0.36	105.60^c^ ± 1.60	0.394^d^ ± 0.23	80.69^b^ ± 1.64
Arid × male	103	24.15^cd^ ± 0.39	123.42^b^ ± 1.71	0.487^d^ ± 0.24	99.26^a^ ± 1.75
Semi-arid × female	147	25.08^bc^ ± 0.32	111.18^c^ ± 1.43	0.424^c^ ± 0.20	86.10^b^ ± 1.47
Semi-arid × male	149	26.54^a^ ± 0.32	128.78^ab^ ± 1.44	0.502^bc^ ± 0.21	102.24^a^ ± 1.47
Dry sub-humid × female	57	24.58^bcd^ ± 0.52	128.86^ab^ ± 2.31	0.394^ab^ ± 0.33	104.28^a^ ± 2.37
Dry sub-humid × male	61	26.15^ab^ ± 0.49	106.96^c^ ± 2.18	0.509^abc^ ± 0.31	80.81^b^ ± 2.23
Humid × female	88	23.42^d^ ± 0.43	110.75^c^ ± 1.90	0.430^a^ ± 0.27	87.33^b^ ± 1.94
Humid × male	107	25.32^abc^ ± 0.38	131.66^a^ ± 1.68	0.514^a^ ± 0.24	106.34^a^ ± 1.72

^a, b, c, d^Values in the same column with different superscripts differ significantly (*P* < 0.05)

*BW* birth weight, *WW* weaning weight, *P-ADG* pre-weaning average daily gain, *P-WG* pre-weaning gain

Agro-ecological zone was a significant (*P* < 0.05) source of variation for weights at birth and weaning, P-ADG and P-WG of Nguni calves (Table 5). The birth weight was highest in semi-arid zone (25.81 kg). Pre-weaning weights of Nguni calves in the semi-arid and humid zones were higher (*P* < 0.05) than those of calves in the arid zone. However, calves of the arid zone and dry sub-humid zones had similar (*P* > 0.05) WW. Similarly, calves in semi-arid, dry semi-humid and humid zones had similar (*P* > 0.05) WW. The P-ADG of calves from all agro-eco zones differed significantly from each other. The WW was highest in humid zone (121.21 kg) and lowest in arid zone (114.51 kg). Calves in humid zone gained more weight than calves in all other agro-ecological zones. The P-ADG was highest in humid zone (0.477 kg/day) and lowest in arid zone (0.438 kg/day).

**Table 5:** Pre-weaning calf performance (kg) of Nguni cattle in different agro-ecological zones of Limpopo Province, South Africa

Source	*N*	BW	WW	P-ADG	P-WG
Arid	217	24.53^b^ ± 0.26	114.51^b^ ± 1.17	0.438^d^ ± 0.17	89.98^b^ ± 1.20
Semi-arid	296	25.81^a^ ± 0.23	119.98^a^ ± 1.01	0.464^c^ ± 0.15	94.17^a^ ± 1.04
Dry sub-humid	118	24.36^b^ ± 0.36	117.91^ab^ ± 1.59	0.450^b^ ± 0.23	92.55^ab^ ± 1.62
Humid	195	25.36^ab^ ± 0.29	121.21^a^ ± 1.27	0.477^a^ ± 0.18	96.83^a^ ± 1.30
Means	826	25.02 ± 0.29	118.40 ± 1.26	0.458 ± 0.18	93.38 ± 1.29

^a, b ,c ,d^Column means with different superscripts differ significantly (*P* < 0.05)

*BW* birth weight, *WW* weaning weight, *P-ADG* pre-weaning average daily gain, *P-WG* pre-weaning gain

Season effect on pre-weaning calves performance of Nguni cattle in Limpopo Province is presented in Table 6. Season had a significant effect on BW, P-ADG and P-WG. Season of birth had no effect (*P* > 0.05) on WW. Summer calving season yields higher WW, P-ADG (0.461 kg/day) and P-WG (94.69 kg) than winter calves. Summer calves gained 2.57 kg more than winter calves.

**Table 6:** Effect of season of birth on pre-weaning calf performance (kg) of Nguni cattle in Limpopo province, South Africa

Season	Birth weight (kg)	Weaning weight (kg)	P-ADG (kg/day)	P-WG (kg)
Summer	24.71^a^ ± 0.1838	119.37^a^ ± 0.8163	0.461^a^ ± 0.1165	94.67^a^ ± 0.8356
Winter	25.33^b^ ± 0.2212	117.43^a^ ± 0.9824	0.449^b^ ± 0.1402	92.10^b^ ± 1.0055

^a, b^Column means with different superscripts differ significantly (*P* < 0.05)

Table 7 shows the effect of agro-ecological zone and season of birth on pre-weaning traits of Nguni calves. In arid and semi-arid areas, the performances of animals born in the summer are better than those of animals born in winter. In sub-humid and humid areas, the performances of animals born in the winter are better than those of animals born in summer.

**Table 7:** Agro-ecological zone and season of birth interaction effects on pre-weaning calf performance (kg) of Nguni cattle in Limpopo province, South Africa

Source	*N*	BW	WW	P-ADG	P-WG
Arid × summer	125	24.10^b^ ± 0.34	116.14^bc^ ± 1.52	0.449^c^ ± 0.22	92.04^abc^ ± 1.56
Arid × winter	92	24.96^ab^ ± 0.40	112.87^c^ ± 1.78	0.424^c^ ± 0.25	87.91^c^ ± 1.82
Semi-arid × summer	182	25.38^ab^ ± 0.28	123.18^a^ ± 1.26	0.478^b^ ± 0.18	97.80^a^ ± 1.29
Semi-arid × winter	114	26.24^a^ ± 0.36	116.78^bc^ ± 1.59	0.441^b^ ± 0.23	90.53^bc^ ± 1.63
Dry sub-humid × summer	66	25.22^ab^ ± 0.47	117.68^abc^ ± 2.10	0.455^ab^ ± 0.30	92.47^abc^ ± 2.14
Dry sub-humid × winter	52	25.51^ab^ ± 0.54	118.14^abc^ ± 2.38	0.443^ab^ ± 0.34	92.64^abc^ ± 2.44
Humid × summer	122	24.132^b^ ± 0.35	120.50^ab^ ± 1.54	0.472^a^ ± 0.22	96.37^ab^ ± 1.58
Humid × Winter	73	24.61^ab^ ± 0.45	121.91^ab^ ± 2.01	0.484^a^ ± 0.29	97.30^ab^ ± 2.06

^a, b, c, d^Values in the same column with different superscripts differ significantly (*P* < 0.05)

*BW* birth weight, *WW* weaning weight, *P-ADG* pre-weaning average daily gain, *P-WG* pre-weaning gain

The interaction between season of birth and sex of calves is presented in Table 8. There was no significant (*P* > 0.05) effect of season and sex of calves’ interaction on BW; however, effects (*P* < 0.05) were observed in WW and P-WG. Male summer calves and winter female calves performed better (*P* < 0.05) than male winter calves and female summer calves in terms of WW and P-WG.

**Table 8:** Season of birth and sex of calves’ interaction effects on pre-weaning calf performance (kg) of Nguni cattle in Limpopo province, South Africa

Source	*N*	BW	WW	P-ADG	P-WG
Summer × females	240	24.74^a^ ± 0.27	110.58^b^ ± 1.18	0.419^a^ ± 0.23	85.85^b^ ± 1.21
Winter × females	161	25.62^a^ ± 0.32	128.20^a^ ± 1.42	0.500^a^ ± 0.20	102.58^a^ ± 1.45
Summer × males	255	24.68^a^ ± 0.25	128.16^a^ ± 1.13	0.504^a^ ± 0.24	103.48^a^ ± 1.16
Winter × males	170	25.04^a^ ± 0.31	106.66^b^ ± 1.36	0.398^a^ ± 0.19	81.61^b^ ± 1.39

^a, b^Values in the same column with different superscripts differ significantly (*P* < 0.05)

*BW* birth weight, *WW* weaning weight, *P-ADG* pre-weaning average daily gain, *P-WG* pre-weaning gain

## Discussion

Agro-ecological zone as described by rainfall, temperature, vegetation type, soil type and topography is a significant source of variation and explains 6.8 and 3.4 % of the variation in pre-weaning and weaning weight, respectively (Bufenning et al. 1982; Dooley 1982; Ronchiotto 1993). Fluctuations in WW, P-ADG and P-WG performance across the agro-ecological zones depict the sensitivity of calves to postnatal stress and can be attributed to nutritional differences between the agro-ecological zones. The findings of this study that wide variation in agro-ecological zone influences any performance traits under study agree with several reports, BW (Burfenning et al. 1982), WW (Dooley et al. 1982; Leighton et al. 1982), P-WG (Treden et al. 1982). These findings are in agreement with the several reports (Wollny 1995; Boitsime 2006; Zindove 2014; de Waal 1990) who deduced that economically important traits in livestock are influenced by variation in the production environment.

The finding that calves in arid zone are lighter in WW, P-ADG and P-WG could be a direct result of poor nutrition, rainfall pattern, veld type, mineral status of the soil and heat stress. However, these areas are characterised by fluctuations in forage quality and quantity resulting in continuous cyclic livestock weight losses and gains which is confirmatory to several reports (DeNise and Ray 1987; Boitsime 2006). The arid and semi-arid zones are subjected to frequent droughts and low rainfall as compared to sub-humid and humid zones. This is in line with arguments put forward by Maciel et al. (2013) that Nguni cattle kept under different environments will perform differently.

Low WW, P-WG and P-ADG in arid zone may also be attributed by exposure of nursing dams to prolonged nutritional stress leading to reduced milk production hence nutrient deprivation in the calves. Variations in WW, P-WG and WW can be a result to differences in milk production between dams which is directly influenced by agro-ecological zone factors such as rainfall, temperature and type of vegetation. This also agrees with several reports (Letholu 1983; Dionisio 1989; Bothma 1993; Erat and Buchanan 2005) that 50 to 70 % of WW, P-WG and P-ADG variation is caused by agro-ecological factors. Arid and semi-arid zone summer calves yielded more weight because they were calved in the season where most rainfall is received (Schulze 1997) which in turn leads to higher milk production by their dams. On contrary, humid and sub-humid zones receive enough rainfall throughout, but their soils have low levels of phosphorus (Hunters and Buck 1992) which may reduce feed intake, reduced weight gain (Gartner et al. 1981) of cattle.

The higher WW, P-WG and P-ADG in humid and dry sub-humid zones calves are attributed by favourable agro-ecological factors which have induced minimal postnatal stress. This could have resulted from the fact that these zones receive enough rainfall for plant or forage growth, and its soils have high level of iron and aluminium which are essential for optimum plant growth (Mohammed-Saleem 1995) resulting in cows producing more milk.

The season of birth shows a significant effect on BW, P-ADG and P-WG of Nguni calves. Melaku et al. (2011) reported that season had an effect on BW of Fogera calves. However, contrary findings by Amsalu (2003), Getinet et al. (2009) and Addisu et al. (2010) reported non-significant effects of season on BW of calves of different breeds (Nguni, Bonsmara, Hereford and Brahman). Summer calves had higher WW than winter calves because they were calved in season where most of the rain is received which is ample for plant growth and therefore milk production by their dams. On contrary, Venter (1977) reported that Nguni winter calves had higher weaning weight.

Fairly constant trends in BW across the sex of calves indicate the ability of the Nguni dams to provide a neutral prenatal environment for calf growth irrespective of the sex of the calves. Similar results had been reported (Maarof and Arafat 1987; Wilson and Traole 1988); however, on the contrary, Melaku et al. (2011) reported that BW of Fogera calves is affected by the sex. Except BW, all other weights between sexes were significantly different where male calves tended to weigh heavier at weaning and have higher P-ADG and P-WG than heifer calves. The sexual dimorphism was evident in pre-weaning growth performance of Nguni calves in which male calves weighed heavier than females. Ebangi (2000) also described that male calves are heavier than heifer calves from birth to weaning and ascribed that these differences are due to differences in their endocrinological and physiological functions, together with increased selection for growth rate on male calves compared to heifer calves. Male calves develop more muscle and less fat than females in summer; fat rather than muscle is advantages in winter (Beebee and Rowe 2008), thus causes the male summer calves and winter calves outperforming the male winter calves and female summer calves.

The mean BW of Nguni calves obtained in the present study is similar to the South African Nguni Breeders Association estimated average BW in extensive system. Similar BW had been reported by Schoeman (1996) and du Plessis et al. (2006) who reported 27 and 26.5 kg BW of Nguni calves. However, different results had been reported; Schoeman (1989), Scholtz et al. (2000) and Collin-Lusweti (2000) reported higher Nguni calves’ BW of 29.1, 36 and 30.3 kg, respectively. The mean WW of the present study is lower to reports by Collin-Lusweti 2000 (135 kg), Scholtz et al. (2000) (215 kg), du Plessis et al. (2006) (161 kg) and South African Nguni Breeders Association of 155 kg estimated WW but still acceptable in extensive grazing system. The P-ADG of this study is lower compared to results reported by Scholtz et al. (2000) (0.873 kg/day), Schoeman (1989) (0.734 kg/day) and du Plessis et al. (2006) (0.659 kg/day) in different regions of South Africa.

## Conclusion

In conclusion, the study revealed that pre-weaning growth performance of Nguni cattle is dependent on agro-ecological zone factors. Nguni cattle should be bred to calve in summer season; given a favourable environment, summer calves will outperform winter calves at its optimum genetic potential. Genotype × environment mechanisms can play a major role in pre-weaning performance of Nguni cattle. The results of this study may indicate the presence of genotype × environment mechanisms, and this stimulated a literature study on the effect of genotype × environment mechanisms on animal production. It was concluded that acceptable levels of growth are achievable from Nguni cattle whilst grazing on natural pastures without nutritional supplements under the different agro-ecological zones of Limpopo province, South Africa.
